# Plateau-Shaped Flexible Polymer Microelectrode Array for Neural Recording

**DOI:** 10.3390/polym9120690

**Published:** 2017-12-08

**Authors:** Jun-Min Kim, Changkyun Im, Woo Ram Lee

**Affiliations:** 1 Medical Device Research Center Medical & Health Device Division, Korea Testing Laboratory, Seoul 52852, Korea; wool001@snu.ac.kr; 2 Dental Research Institute, Seoul National University School of Dentistry, Seoul 03080, Korea; oingah@nate.com

**Keywords:** fabrication, multielectrode array (MEA), PDMS, PDMS etching, plateau-shaped electrode, recessed electrode, spinal cord signal recording, underexposure

## Abstract

Conventional polymer multielectrode arrays (MEAs) have limitations resulting from a high Young’s modulus, including low conformability and gaps between the electrodes and neurons. These gaps are not a problem in soft tissues such as the brain, due to the repopulation phenomenon. However, gaps can result in signal degradation when recording from a fiber bundle, such as the spinal cord. Methods: We propose a method for fabricating flexible polydimethylsiloxane (PDMS)-based MEAs featuring plateau-shaped microelectrodes. The proposed fabrication technique enables the electrodes on the surface of MEAs to make a tight connection to the neurons, because the wire of the MEA is fabricated to be plateau-shaped, as the Young’s modulus of PDMS is similar to soft tissues and PDMS follows the curvature of the neural tissue due to its high conformability compared to the other polymers. Injury caused by the movement of the MEAs can therefore be minimized. Each electrode has a diameter of 130 μm and the 8-channel array has a center-to-center electrode spacing of 300 μm. The signal-to-noise ratio of the plateau-shaped electrodes was larger than that of recessed electrodes because there was no space between the electrode and neural cell. Reliable neural recordings were possible by adjusting the position of the electrode during the experiment without trapping air under the electrodes. Simultaneous multi-channel neural recordings were successfully achieved from the spinal cord of rodents. We describe the fabrication technique, electrode 3D profile, electrode impedance, and MEA performance in in vivo experiments in rodents.

## 1. Introduction

A common goal of physiologists and bioengineers is to interface with and elucidate the properties of excitable cells within intact tissues [1]. For example, a brain–computer interface (BCI) couples the nervous system to a device that can either stimulate the tissue or record neural activity, or perform both in a closed-loop system [2]. A BCI typically includes a computer for analyzing signals and an electrode for stimulating or recording a neural signal [2,3]. The electrode is a transducer that converts the activity of an ionic current into an electrical potential, so it directly affects the quality and reliability of the BCI.

Electrodes can be divided into two types: Non-invasive and invasive. Although non-invasive techniques have the advantage of being safe, the spatial resolution is generally lower than that of invasive methods [4,5]. The signal acquired from a non-invasive electrode includes the effect of the activity signal of neuroglia surrounding the stimulated neurons, rather than directly the activity of the neurons being stimulated. However, invasive electrode technologies have higher spatial resolution, and with the rapid growth of computing power, can process a large amount of information simultaneously through a multielectrode array (MEA) [6,7]. MEAs can be classified as either probe-type or planar-type. The probe type is commonly made of rigid materials, such as glass, silicon, and metal, in order to penetrate the tissue surface [7,8]. Inevitably, during the insertion process, a wound occurs and, after insertion, damage to the electrode surrounding the tissue occurs due to the friction caused by the micro-movement of the electrode. Due to the immune rejection and inflammatory response, long-term experiments by using a probe-type electrode are difficult [9,10].

To overcome this drawback, rigid planar MEAs have been developed to interface with the surface of tissues [11]. Glass or silicon wafers are mainly used as substrate materials; particularly, glass wafers are widely used with transmission microscopy. If the signals from the deep part of the neural tissue are to be recorded or the deep part is to be stimulated, a probe-type MEA is required. If the region of interest is close to the surface of the tissue, a rigid planar MEA can be a good alternative in terms of biocompatibility. However, a rigid planar MEA cannot closely follow the curvature of the tissue, which is a disadvantage.

In order to improve the ability of MEA to interface closely and effectively with the tissue surface, MEAs have been fabricated on flexible substrates such as polyimide, parylene, and silk [12,13,14,15]. The rigid planar MEA is commonly used in vitro, while the flexible planar MEA is used widely in vivo because it can cover the complex surface of tissue well. However, as parylene and polyimide are more rigid than neural tissues, micro-movement of flexible planar MEAs can cause tissue injury. In the case of silk electrodes, the electrode location cannot be adjusted after the initial positioning [15].

The PDMS polymer has been used to solve these problems [16,17,18,19,20]. As the Young’s modulus of PDMS is similar to that of neural tissues (approximately 1.0 MPa) [13,17,20], tissue damage is reduced compared to other polymers. In addition, the location of PDMS-based MEAs can be adjusted easily during the experiment, due to good conformability [17,19,21,22]. In spite of these merits, PDMS has not been widely used as a substrate material because it is difficult to apply existing semiconductor processing techniques. The PDMS surface has low surface energy, so it is difficult to deposit metal on it. As it is chemically stable, wet/dry etching is difficult [22,23].

So far, the all-flexible planar MEAs that have been developed have a similar structure, regardless of the type of substrate. First, metal wires are fabricated on a flexible substrate, and after encapsulating the sample using an insulator, electrodes are manufactured by etching the insulator above the wire [12,16,21]. These manufacturing processes always generate a recessed structure over the electrode, which is surrounded by the insulator. As this recessed structure increases the distance between the electrode and the neuron, recording and stimulating performances are degraded. Air trapping phenomena can also occur in this area, disturbing the electrode–neuron interface. In order to prevent air trapping, oxygen plasma treatment has been commonly used [18], but with time, the surface characteristics of PDMS change from hydrophilic to hydrophobic. Therefore, this method is not a permanent solution, and new technology is needed.

In this paper, we propose a new fabrication process to manufacture a PDMS-based planar MEA that does not generate a recessed structure, and instead produces a plateau-shaped electrode. Moreover, we measure the physical characteristic and impedance of the developed MEA, and evaluate air trapping. Finally, we evaluate the performance of the proposed MEA by recording neural activity in vivo in the spinal cord of an anesthetized rodent, stimulated by touching the limb and pelvic regions.

## 2. Methods

### 2.1. Electrode Structure and Fabrication

**Figure 1:**
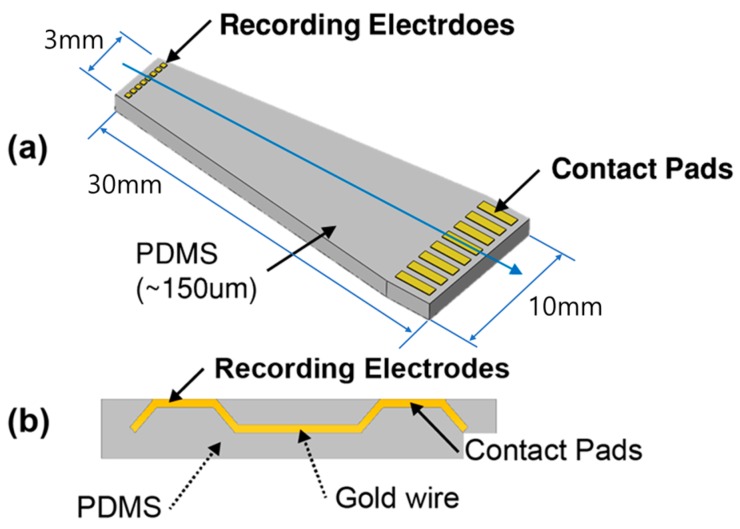
3D schematic of the electrode array. (**a**) Schematic of a polydimethylsiloxane (PDMS)-based multielectrode array. (**b**) Cross-section along the blue arrowed line in (**a**).

Figure 1a shows a 3D structural view of the PDMS-based plateau MEA. A plateau MEA has a total of 8 electrodes and requires an additional interface for connecting MEA and recording equipment. The method of connecting electrodes to external equipment using epoxy glue and soldering was common, but gold thin films on PDMS can be easily broken by external force. The zero-insertion force (ZIF) connector eliminated the need to use epoxy glue or soldering. The ZIF connector (FH12-16S-0.5SH, HIROSE, Tokyo, Japan) was used a device of 16-pin with 0.5 mm pitch. The developed MEA has 8 pads, and we designed the pad size that one pad can use two ZIF pins. We designed the interface pad with a width of 700 μm and a pitch of 1 mm. The electrodes, wires, and interface pads of the zero-insertion force (ZIF) connector are made of gold, and the base material of the electrode is made of PDMS. Figure 1b shows the electrode cross-section. The thickness of the MEA is approximately 150 μm, and thickness can be controlled easily through the PDMS spin-coating speed. The thickness of the substrate inserted into the ZIF connector should be at least 300 μm. Thus, a plastic plate having a thickness of 200 μm was placed behind the MEA and used. The electrode, pads, and wires are parts of a single body, and only the electrode connecting to the neurons and the pad computer interface are exposed to air, as shown in Figure 1b. The plateau electrode structure not only enables the electrode to make solid contact with the neurons, but also improves electrode performance by preventing air from becoming trapped between the electrode and tissue. Based on these characteristics, after attaching the MEA on a brain or spinal cord, we could adjust the location of MEA to optimize the biological signal recorded.

**Figure 2:**
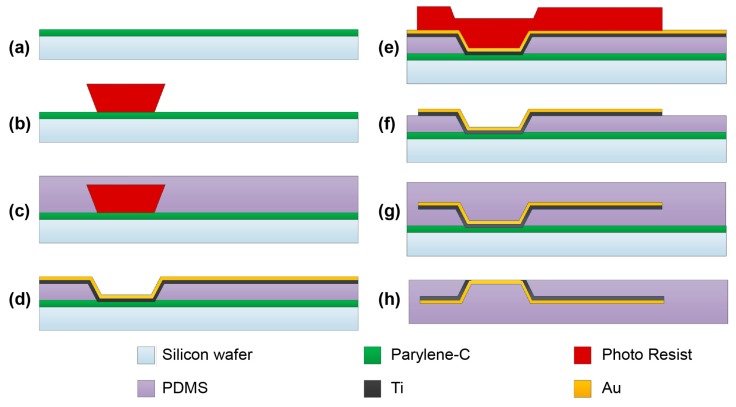
Schematic of fabrication process for a PDMS-based plateau electrode array.

The sequential processes for fabricating the MEA are shown schematically in Figure 2, and described in detail below. First, a cleaned silicon wafer was coated with parylene (Parylene-C, CNC Chemical, San Clemente, CA, USA) to obtain weak bonding between the silicon wafer and PDMS film. This prevented the metal from joining with the silicon wafer, and helped to strip the MEA from the wafer in the final process. A parylene layer of 3 μm was deposited using parylene deposition equipment (PDS2010, SCS Inc., Glendale, CA, USA), as shown in Figure 2a. A sacrificial post was subsequently built on the sample via photolithography, as shown in Figure 2b. The negative photoresist (DNRL300-40, DONGJIN SEMICAM Co., Seoul, Korea) used in this research was spin-coated at 1300 rpm for 20 s, and a thickness of 7 μm was achieved. Next, a double-coating scheme was used to achieve a photoresist layer thickness of 20 μm. The sample obtained after the second coating was exposed at 25 mW/cm^2^ for 18 s using exposure equipment (MA6 Aligner, SUSS MicroTec Group, Corona, CA, USA). Subsequently, in order to adjust the inclination of the sacrificial post, we placed a 160-μm transparent polyethylene terephthalate (PET) film between the sample and mask. The inclination of the post could be controlled by regulating the thickness of the PET film and the exposure time. The sample was subsequently treated with oxygen plasma by using reactive ion etching (RIE) (RIE 80 Plus, Oxford Instruments, Abingdon, UK) in order to increase the adhesion force between parylene and PDMS. PDMS (Sylgard R184, Dow Corning, Co., Midland, MI, USA) was spin-coated onto the sample at 1500 rpm for 60 s, as shown in Figure 2c. The sample was subsequently cured in an 80 °C oven for 2 h. After curing, the PDMS layer was wet-etched using a mixture of tetrabutylammonium fluoride (TBAF, Tokyo Chemical Industry, Tokyo, Japan) and *n*-methylpyrrolidone (NMP, Tokyo Chemical Industry, Tokyo, Japan) until the sacrificial post was revealed. The PDMS etchant consisted of TBAF and NMP mixed at a ratio of 1:10 (*v*/*v*) [24]. After PDMS etching, the photoresist residue on the electrodes and pads was cleaned using NMP. The sample was subsequently cleaned using de-ionized (DI) water, and sonication was performed for 5 min in DI water. Before depositing gold onto the PDMS in the hole regions, 2-hydroxyethyl methacrylate (HEMA) was polymerized on the PDMS and parylene film by using oxygen plasma to increase metal adhesion. The polymerization method consisted of three steps: The first step consisted of increasing the surface energy of the polymer via oxygen plasma, which helps HEMA combine with the PDMS and parylene [25]. The parameters of the RIE equipment for the oxygen plasma treatment were as follows: a pressure of 75 mTorr, oxygen flow rate of 20 sccm, and power of 100 W; these parameters were the same in all steps.

In the second step, HEMA was spin-coated onto the sample at 1500 rpm for 30 s. The oxygen plasma treatment was repeated in the third step. Thus, we could graft an amphiphilic HEMA film onto the PDMS surface. After surface treatment, titanium with a thickness of 300 Å was deposited on the sample using an electron gun evaporator (ZZS550, MAESTECH, Pyeongtaek, Korea) to increase the adhesion between the gold and PDMS. Subsequently, a gold layer with a thickness of 3000 Å was deposited. Although gold was used as the electrode material in this study, according to recent trends in the manufacture of electrodes, Pt, IrO2, or TiN can also be used. Titanium was deposited at 3 Å/s until a thickness of 300 Å was obtained, and gold was deposited at 2 Å/s until a thickness of 3000 Å was obtained, as shown in Figure 2d.

In order to pattern the gold layer, photoresist was coated under the same conditions as described earlier, and exposed for 20 s without the PET film, as shown in Figure 2e. The gold layer was etched by a solution of iodine and potassium iodide at room temperature. After titanium was revealed, it was etched using a buffered oxide etcher (BOE), as shown in Figure 2f. PDMS was spin-coated for strong bonding after treating the PDMS surface by oxygen plasma. The encapsulating PDMS layer was manufactured by spin-coating at 700 rpm for 60 s, as shown in Figure 2g. The thickness of the PDMS encapsulation layer was approximately 100 μm, depending on the spin-coating speed. The outline of the MEA was cut by a Nd:YVO_4_ laser (Series 3500 UV Laser System, DPSS Lasers, Inc., Santa Clara, CA, USA), and the process conditions were as follows: power of 2.4 W, speed of 2 mm/s, wavelength of 10 μm, and frequency of 20 kHz. The sample was soaked in 70% ethanol solution for 30 min to weaken adhesion between the parylene and silicon wafer. The parylene layer above the gold electrode was easily etched using the RIE system for 10 min under an oxygen flow rate of 20 sccm, pressure of 350 mTorr, and power of 65 W, as shown in Figure 2h. After removing the parylene, the Ti layer was etched using BOE, and the sample placed in DI water for 1 day to remove contaminants.

### 2.2. Device Characterization

#### 2.2.1. Electrode Profile

The most important aspect of the electrode manufacturing was ensuring that the holes in the PDMS film had a gentle slope. The reasons to fabricate holes with a gentle slope can be explained as follows: As the electrode of the RIE system is commonly oriented perpendicular to the wafer, the plasma has a vertical direction. If the wall of the PDMS hole is vertical to the wafer, plasma processing does not perform as well. There are many ways to deposit the metal, and categorized in order of step-coverage criteria, sputtering, electron-gun, and thermal evaporation methods all exhibited excellent coverage characteristics. If we were to deposit metal on a vertical wall using a thermal evaporator, the metal would not uniformly cover the wall, but rather accumulate in the corners. In order to solve these problems, holes with a gentle slope are necessary. Such holes facilitate the surface treatment of PDMS, and enable various metal depositions on the sample.

A negative photoresist film of thickness 20 µm was manufactured to produce a variety of inclined PDMS holes. While adjusting the contact conditions of the photomask and exposure energy, we measured the slope angle of the sacrificial post. The photomask contact conditions were approximate, soft, and hard, and the exposure time was tested at intervals of 2 s over 12–20 s. We manufactured sacrificial posts using the photolithographic conditions obtained from the above experiment, and coated the PDMS film such that it was thicker than the height of the post. Bare, fluorocarbon (FC), and HEMA-coated wafer were prepared to obtain a PDMS film of proper thickness. Uncured PDMS was diluted in tert-butyl alcohol (TBA), and 50% and 25% solutions (*w*/*w*) were prepared in order to determine which characteristics depended on concentration [26]. We spin-coated PDMS on the prepared wafer for 60 s at 500, 1000, 2000, and 4000 rpm, and measured the film thickness using Alpha-Step (Nanospec AFT/200, KLA-TENCOR, Milpitas, CA, USA). The alpha-step equipment was developed for measuring level differences on the sample surface. When the stylus is scanned the sample surface, it senses the pressure caused by a level difference, and measures the height of the level. The stylus was a diamond needle, which had a diameter of 10 µm, stylus force of 17.3 mg, scan speed of 20 μm/s, sampling rate of 50 Hz, and vertical resolution of 25 Å.

TBAF solutions of four types (6, 9, 16, 25% (*v*/*v*)) were prepared for measuring the etching rate of PDMS. After an aluminum tape was attached to the PDMS sample, it was soaked in TBAF solution for 20 min. Subsequently, the tape was removed from the PDMS sample, and the etching rate of PDMS was evaluated using alpha-step.

The 3D profiler μSurf (NanoFocus, Oberhausen, Germany) was used to analyze the 3D structure of the electrode. The 3D profiler could measure the 3D profile without contact using confocal multi-pinhole (CMP) technology; however, due to the measurement mechanism using reflected visible light, we could not measure transparent materials such as glass and thin PDMS. In order to verify the 3D hole profile of the PDMS film, we measured the electrode region after gold deposition.

#### 2.2.2. Surface Treatment

PDMS exhibits poor adhesion to metal, and the hydrophobic surface had to be modified to a hydrophilic surface by using an oxygen plasma treatment. However, this method is not permanent because the hydrophilic PDMS surface gradually turns hydrophobic over time. If the metal deposition and plasma treatments occur more than 24 h apart, it would be difficult to use an oxygen plasma process. Instead, in this study, we used a three-step surface modification process to treat the PDMS surface. The first step included modifying the PDMS surface using an RIE system, which enables the HEMA to spread out stably. The second step consisted of applying the HEMA coating, and the third step consisted of grafting the HEMA polymer using oxygen plasma. Each sample was stored 1 h and 48 h at 25 °C before gold was subsequently deposited. Furthermore, HEMA-treated samples were maintained each 1 day and 7 days at 25 °C before gold was deposited. The Scotch tape test was used to test the adhesion of the gold film to PDMS [27]. The experiments were conducted with two types of tapes that have different adhesive forces—one having an adhesion force of 2.5 N/100 mm (810, 3M Inc., Maplewood, MN, USA) was denoted as tape 1, and the other having an adhesion force of 14 N/100 mm (483, 3M Inc., Maplewood) was denoted as tape 2. Pieces of adhesive tape were placed on the gold film and a pressure of 64.5 gf/cm^2^ was applied for 5 min. To apply uniform pressure on the adhesive tapes, a slide glass of weight 500 g was placed on the tape. When the adhesive tapes were released from the PDMS, a segment of the gold film could be detached from the PDMS along with the tape, depending on the adhesive force between the gold film and PDMS.

#### 2.2.3. Electrode Impedance Measurement

The electrical properties of the electrodes were evaluated using impedance spectroscopy. The recessed electrode structure was placed 12 μm below the surface of the MEA, and the other electrode was mounted superficially on the surface of the MEA. In the case of the recessed electrode, due to the hydrophobic surface of PDMS, oxygen plasma modification should be performed to prevent air trapping. We used the method and setup reported in Ref. [28] to measure the electrode impedance. One MEA that we used has eight electrodes of the same size, and ten MEAs have a total of 80 electrodes. By measuring the impedance five times from 80 electrodes, a total of 400 values can be obtained. The impedance result was obtained by averaging these values. The impedance values of recording sites (each site having dimensions of 130 μm × 130 μm) were determined in a phosphate-buffered saline solution (PBS solution, Gibco #10010, Invitrogen Life Technologies, Waltham, MA, USA) at room temperature using an impedance analyzer (IM6e, Zahner-Elektrik GmbH & Co., Kronach, Germany). A three-electrode arrangement was used, with an Ag/AgCl reference electrode (K0260, ATFrontier, Anyang, Korea) and Pt counter electrode (RDE0021, ATFrontier, Anyang, Korea). A 5 mV RMS sine wave was applied to the electrodes with the frequency ranging logarithmically from 100 Hz to 100 kHz.

#### 2.2.4. Surface Contact Performance

A surface contact experiment was performed to examine whether the two types of electrode were properly attached to the nervous system in solution [18]. After the MEAs were immersed in DI water in a petri dish, a cover glass (24 mm × 40 mm × 0.2 mm) was attached to the electrode surface, and subsequently removed. After attaching the two types of MEA to the cover glass in the same manner, the electrode was inspected using an optical microscope.

### 2.3. In Vivo Experiments

Female rats (Sprague-Dawley, Taconic, Köln, Germany) were used for the experiment (Protocol number: Hallym2016-58). The animals were wild-type and had no history of surgical treatment or disability. Furthermore, they were bred in isolation from external environment by automated equipment, and water and food were provided every 12 h. They were anesthetized using an intramuscular injection with Zoletil (Virbac SA, Carros, France) 0.2 mL and Rompun (Bayer Pharmaceuticals, Berlin, Germany) 0.1 mL. After inserting the catheter into the urinary tract of the anesthetized rats and emptying the bladder, a laminectomy was performed from L2 to L1 of the spinal cord. After disinfecting from the 13th thoracic vertebrae to the 3rd lumbar, the spinal cord was exposed by removing the bump via laminectomy. The dura mater of spinal cord was removed, and the spinal cord was fixed using stereotaxic equipment (Model 1040, David Kopf Instruments, Tujunga, CA, USA). First, the neural signal was recorded by an 8-channel tungsten wire electrode. The sampling rate of the recorder (Micro1401-3, CED, Salford, UK) was set to 25 kHz, the gain was 1 k, and frequency range of the band-pass filter was 0.3 to 1000 Hz. We searched for places where the neural signal was generated by pressure stimulation using a tungsten electrode. After selecting a location where the firing rate was high, the neural signal was recorded. Subsequently, using the proposed MEA with 8-channel, we recorded the signal in the same location under the same conditions. The pad area of the proposed MEA is connected to the recorder equipment using the ZIF connector. The recorder consists of amplifier and data acquisition device. The recorder connected to the computer and records the signal in real time using USB interface. A constant-pressure stimulus was applied to the pelvic and limb regions using a custom-built touch-test sensory evaluator. A stimulation sequence was composed of 30 s of rest, 30 s of stimulus, and 30 s of rest, and the experiment was repeated 2–3 times. We recorded both single-unit signals and the local field potential using the PDMS electrode.

## 3. Results and Disscussion

**Figure 3:**
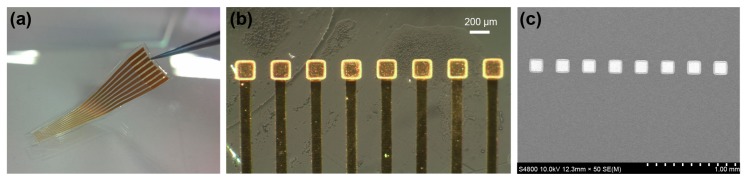
Images of fabricated PDMS-based plateau MEA. (**a**) The overall appearance of the MEA. The sacrificial photoresist post of this sample was spin-coated twice at 1300 rpm for 20 s. The thickness was 20 μm and the depth of the PDMS hole was 11 μm. (**b**) Optical microscope image of the plateau microelectrodes. The bright region around the electrode is the gold film on the wall of the PDMS hole. (**c**) SEM image of the plateau microelectrodes. As the gold wires are inside the PDMS, we could not observe them using SEM. The electrodes were deposited with dimensions of 100 μm × 100 μm, but the SEM measurements show dimensions of 130 μm × 130 μm. The increase in electrode size is caused by the addition of the 160 μm PET film and the shrinking and etching of PDMS as a result of the NMP and TBAF solutions.

The overall appearance and the electrode region of the fabricated PDMS-based plateau MEA are shown in Figure 3. The dimensions of the gold electrodes were 130 μm × 130 μm, and the electrodes were mounted superficially on the PDMS surface. The experiments characterized the electrode 3D profile, electrode impedance, contact performance, and in vivo performance. The following sections describe these experimental results.

### 3.1. Electrode Profile

**Figure 4:**
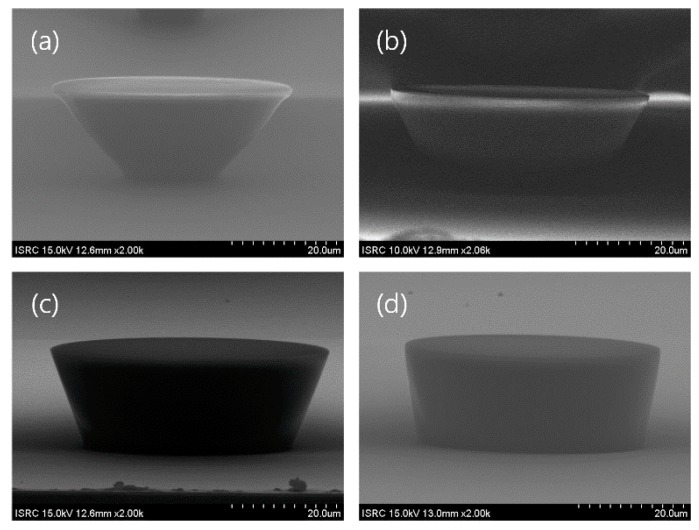
SEM images of the inclined sacrificial post under different exposure conditions. The height of the photoresist post was 20 μm, and the pattern of the photomask was a circle with a diameter of 50 μm. (**a**) A 160 μm PET film was placed between the wafer and photomask, and the exposure time was 14 s at 25 mW/cm^2^. The angle of the resulting post was 50°, obtained by averaging 9 individual posts from five rounds of fabrication. (**b**) The same PET film was used, with an exposure time of 14 s. The measured angle was 63°. (**c**) Soft contact was used, the exposure time was 14 s, and the angle was 72°. (**d**) Hard contact was used, the exposure time was 14 s, and the angle was 83°.

The proposed plateau MEA features microelectrodes with a plateau structure. While manufacturing these electrodes, the most important aspect was the manufacture of the sacrificial post, which has an inclined wall, as the shape of the cured PDMS was determined by the shape of the sacrificial posts. Furthermore, both the spin-coating speed of the uncured PDMS and the etching time of the cured PDMS affected the 3D profile of the electrode. While manufacturing the sacrificial posts, the slope of the wall depended on two factors—the gap between wafer and photomask, and the exposure energy. Based on the gap between wafer and photomask, photolithography conditions were classified as approximate, soft-contact, hard-contact, or vacuum-contact. The larger the gap between sample and photomask, the larger the effect of optical diffraction; therefore, the gap was a factor that controlled the inclination angle of the post wall. Sufficient time was required when the UV light reached the lower part of the photoresist film, and we could control the amount of UV light absorption in the upper and lower sides by adjusting the exposure time. In the case of a negative photoresist, if the exposure time is reduced, the upper part of the photoresist film absorbs more energy than the lower part. Consequently, the lower region of photoresist is more developed than the upper region. Figure 4 shows scanning electron microscope (SEM) images of the inclined sacrificial post according to the exposure conditions. We demonstrated that the sidewall slope of the sacrificial posts could be controlled by applying the PET film and adjusting the UV light exposure time, as shown in Figure 4. The photomask employed in this study had a circular pattern of diameter 50 μm. By comparing Figure 4a,b, or Figure 4c,d, it can be observed that the longer the exposure time, the larger the slope angle. Comparing Figure 4a,b, we could observe that the PET film results in a decrease of slope angle. Through the above results, it can be observed that a larger gap between wafer and photomask or a shorter exposure time can both reduce the slope angle.

**Figure 5:**
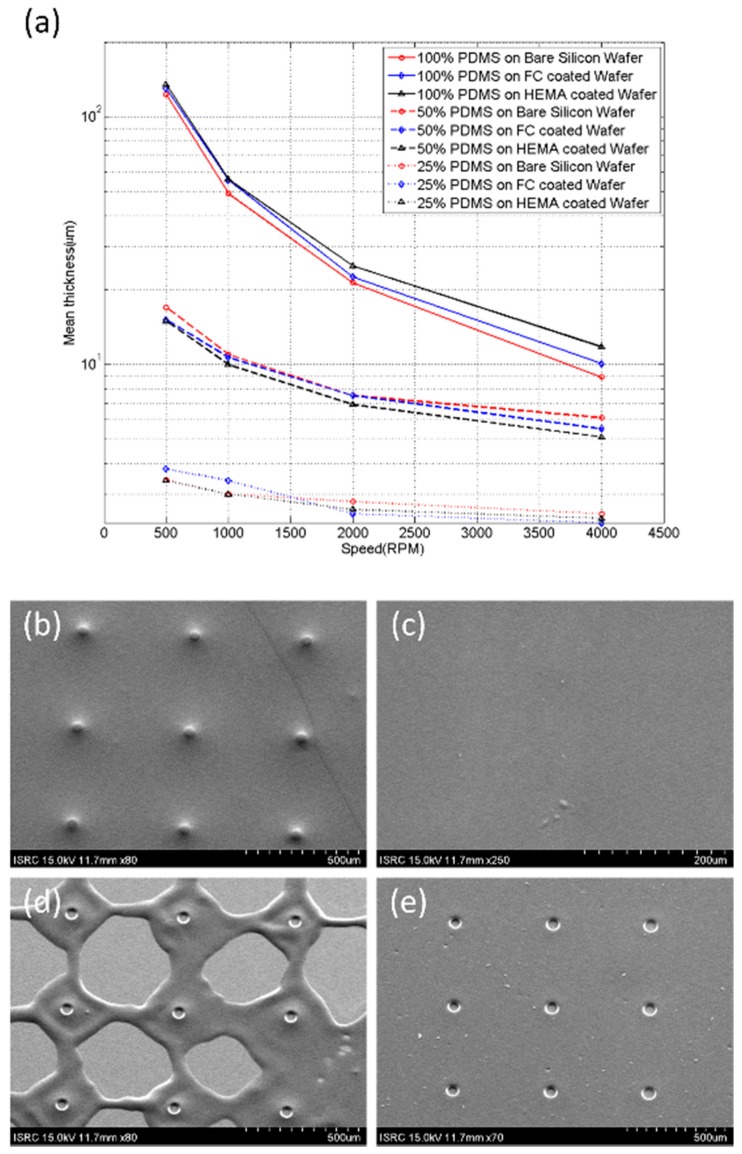
Characterization of PDMS spin-coating. (**a**) The thickness of PDMS film depended on spin-coating speed. The spin-coating speeds were 500, 1000, 2000, and 4000 rpm for 60 s. Each point on the plot corresponds to measurements on 25 samples from 5 rounds of fabrication. (**b**–**e**) SEM images measuring the uniformity of the PDMS film. (**b**) Uncured PDMS spin-coated with a thickness of 10 μm on a photoresist post with a height of 20 μm. (**c**) Uncured PDMS spin-coated with a thickness of 40 μm on a photoresist post with a height of 20 μm. The photoresist posts are not revealed on the surface due to the thick PDMS film. (**d**) Cured PDMS of (**b**) etched by 9% TBAF solution for 4 min. The PDMS in the nearby and far posts were thick and thin, respectively. Hence, we could not obtain a uniform PDMS film. (**e**) Cured PDMS of (**b**) etched by 9% TBAF solution for 10 min. Uniform PDMS film with holes could be obtained.

We measured how the thickness of the PDMS film depended on the spin-coating speed, viscosity, and type of substrate. The film thickness of cured PDMS depended on the spin-coating speed and viscosity, as shown in Figure 5a. In previous studies, the PDMS film was manufactured to be as thin as possible by spin-coating on posts with a height of 20 μm [21]. This method had a low yield, because PDMS residue was formed on the sacrificial posts, as shown in Figure 5b. We attempted to remove the PDMS residue on the posts by using a wet-etching process; however, the non-uniform thin PDMS film was torn frequently, as shown in Figure 5d. This problem could be solved by applying uncured PDMS thicker than the photoresist posts, as shown in Figure 5c. Based on the results of Figure 5a, uncured PDMS was spin-coated on the inclined post with a thickness of 40 μm to obtain good uniformity over 20-μm posts. After etching the cured PDMS and removing the photoresist posts using NMP, a PDMS film with holes could be obtained; however, if the uniformity of the PDMS film was poor, the setup of the etching process was difficult, as shown in Figure 5b,d. The PDMS film with holes could be obtained using a uniform PDMS film, as shown in Figure 5e. Methods for etching the PDMS include dry and wet methods. As dry-etching was very slow (0.1 μm/min) [24], we used the wet-etching method because tens of micrometers of PDMS had to be removed. The wet-etching solution was a mixture of TBAF and NMP, and the PDMS etching rates at room temperature are shown in Table 1. The 25% TBAF solution exhibited a high etching rate, but PDMS slurry leftover after etching was a drawback [23]. While the 6% solution exhibited a low etching rate, the etching time and exposure time to NMP were prolonged, which could adversely affect the PDMS film [29].

The PDMS etching method has been developed in various ways [17,18,21,23,24]. A method that uses sacrificial posts should be employed in order to adjust the inclination of the PDMS hole [18]. Therefore, we manufactured sacrificial posts with various inclinations using underexposure and approximate exposure techniques. After obtaining sacrificial posts, the standard method to obtain a thin PDMS film with holes would be to spin-coat PDMS onto the sample at very high speed. However, if this method was used, the PDMS film would be thick near the sacrificial posts and thin far from the posts; thus, the uniformity of film would be degraded. Additionally, PDMS residues on the sacrificial pillar caused problems [23]. To solve these two problems simultaneously, after coating the PDMS to be thicker than the sacrificial posts, the overall PDMS film was wet-etched until the posts were revealed.

**Figure 6:**
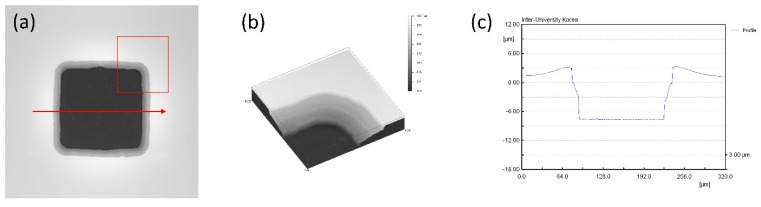
3D rear profile images of plateau electrodes. (**a**,**b**) Wide field optical microscope images of an electrode. (**a**) The gray rectangle band shows the slope of the electrode. (**b**) An enlarged 3D projection of the region in the red rectangle of the image in (**a**). We can observe the gently sloping PDMS wall, which has an inclination angle of 40°. (**c**) 2D profile of the electrode obtained along the red line in (**a**). One side of the electrode is approximately 130 μm, and the depth of the electrode is approximately 11 μm.

Figure 6 shows the 3D profile measurements of the electrode. In order to determine and measure the key fabrication parameters of the plateau electrode structure, an analysis of the electrode profile was necessary. The 3D profiler could not detect a transparent structure due to the use of white visible light; thus, the electrode profile was measured after gold deposition. Figure 6a illustrates the rear region of electrode, and shows a gray rectangular band of inclined wall. The red rectangle in Figure 6a is separated into full sheets (Figure 6b in order to view the 3D structure of the electrode intuitively. Figure 6c illustrates a cross-section image of the red line in Figure 6a, and shows a 2D profile of the electrode area. The depth of the electrode was approximately 11 μm, and the length of the base was approximately 16 μm; thus, we could calculate the angle of sloped wall to be approximately 40°. The depth of the electrode was determined by the etching time of PDMS and the initial height of the sacrificial posts. The mask pattern size for producing an electrode was 100 μm, but the measured size of the electrode was approximately 130 μm, which was extended by 30%. These errors could be caused by underexposure, or approximate exposure, in photolithography, or the PDMS film could also be shrunk by NMP exposure during the PDMS etching. Therefore, in order to obtain an electrode of diameter 100 μm, a photomask of diameter 80 μm should be used.

**Figure 7:**
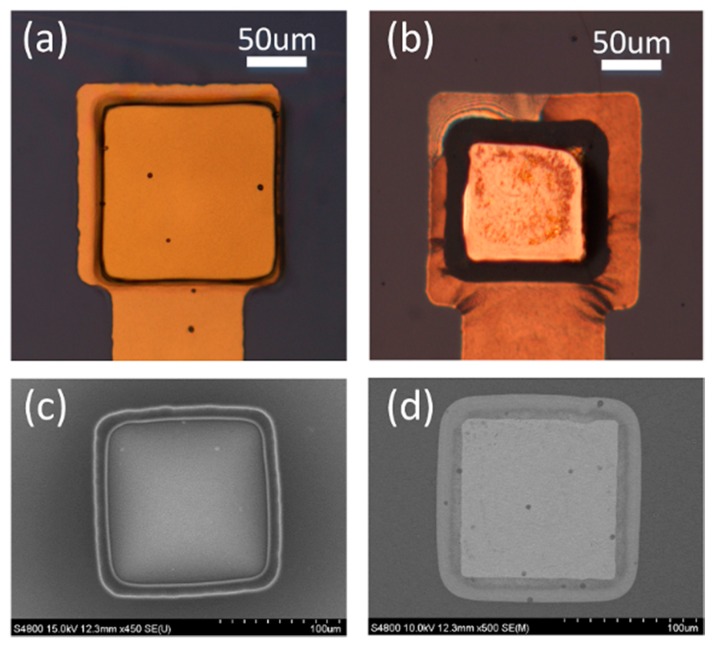
Optical and SEM images of a recessed electrode and a plateau electrode. (**a**) Enlarged image of the recessed electrode obtained using an optical microscope. Metal wires can be seen to pass under the PDMS film. (**b**) Plateau electrode. The dark band shows the PDMS slope connecting the electrode and wire. (**c**) SEM image of recessed electrode. The inclined holes can be observed on the PDMS film. (**d**) SEM image of plateau electrode.

Using this structure, a gold film could be stably deposited on the wall of PDMS holes via electron-gun equipment. Figure 7 shows enlarged images of the two types of electrode using an optical microscope and SEM. The gold electrode of the conventional recessed electrode was placed under the PDMS film in Figure 7a, and an image of the inclined PDMS hole is shown in Figure 7c. The gold was deposited along the gently sloped PDMS surface and the electrode was connected to the wire, as shown in Figure 7c. Moreover, we could observe a gray band on the edge of the electrode. When performing oxygen plasma treatment to increase the adhesion force between parylene with PDMS, parylene was slightly etched, resulting in the gray band was observed in SEM. This problem could be mitigated by decreasing the plasma processing time.

### 3.2. Surface Treatment

The surface of PDMS was modified using oxygen plasma treatment and HEMA-grafting in an RIE system. HEMA was coated on oxygen-treated PDMS to facilitate the attachment of HEMA on the PDMS surface. The oxygen plasma-treated films demonstrated a very high degree of hydrophobic recovery, whereas HEMA coated onto oxygen-treated PDMS showed only a small decree of reconstruction [25]. To assess these properties, a gold thin film was deposited on the PDMS and the adhesion between the thin gold film and pretreated PDMS was tested by an adhesion test. The results from two different adhesive tapes are shown in Table 2.

After the oxygen plasma-treated PDMS was stored for 24 h at room temperature, both tests failed due to the reoccurrence of hydrophobicity. However, samples stored for only 1 h passed both tests. HEMA-treated PDMS samples were stored for 1 h, 24 h, and 7 days in room temperature. In this case, all samples passed the gold adhesion test. Thus, HEMA processing maintained the hydrophilic surface of the PDMS for a long time, and facilitated the follow-up processes.

### 3.3. Electrode Impedance Measurement

The equivalent circuit model of electrode and electrolyte interface consists of solution resistance, a constant phase element and the charge transfer resistance [30]. The constant phase element and the charge transfer resistance are connected in parallel and the solution resistance is connected in series. These factors are the theoretical values typically used to represent the electrode–electrolyte impedance [31]. The charge transfer resistance and constant phase element are factors influenced by the size and structure of the electrode, and the solution resistance is the resistance due to the solution between the working electrode and the counter electrode. It can be assumed that solution resistance and the constant phase element are the same because the fabricated electrodes have been exposed to the same process.

**Figure 8:**
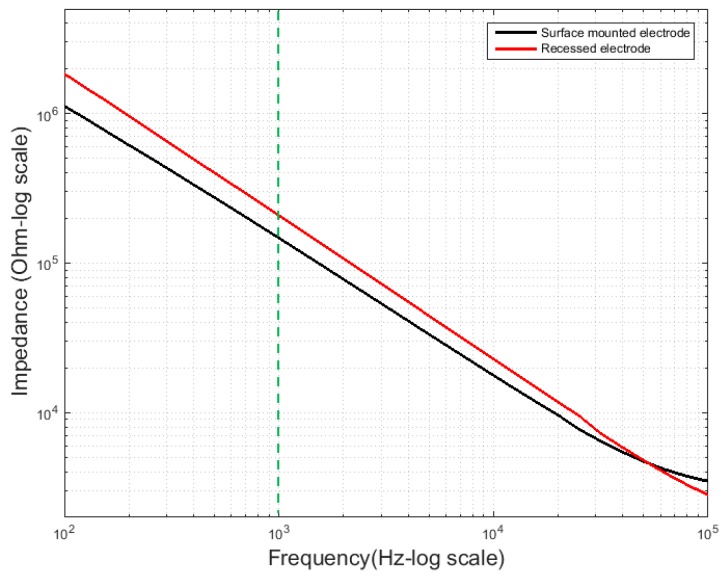
Electrode impedance measurement. Five measurements of electrode impedance on the eight individual electrodes of 10 MEAs are considered. The average electrode impedance curves rely on the electrode area and depth.

Figure 8 shows the average electrode impedance curves corresponding to the two types of electrodes. The average impedance values at 1 kHz were 150 kΩ for the plateau type and 220 kΩ for the recessed type. This was close to the required impedance range for a single-unit recording neural probe [27,32]. The electrode size of the recessed type was 108 μm × 108 μm, and that of the plateau type was 130 μm × 130 μm. When the electrode is in contact with a living tissue composed of the electrolyte, an electrochemical double layer is formed between the electrode and the electrolyte, and this phenomenon can be quantified using impedance [8,9]. Therefore, the impedance largely depends on the area of the electrode; ignoring the material and deposition condition of the electrode, the impedance of the electrode is inversely proportional to the electrode area. The area of the plateau electrode was approximately 1.45 times larger than that of the recessed electrode, and the difference in the impedance values increased because the electrodes were located at a depth of 12 μm from the surface. Assuming that the thickness and roughness of the gold were identical, the impedance difference between the two electrodes may be considered to be caused by the area and depth of the electrodes. If the area of recessed type electrode is converted into the same area to plateau electrode, the impedance of recessed electrode can be about 151.7 kΩ. Therefore, it can be seen that an additional impedance of about 1.7 kΩ occurs due to the depth difference of about 12 μm. Because the impedance values are similar, the conductivity of the two MEA types can be considered to be similar to each other. However, in order to support this opinion, additional studies are required through the same electrode size and various recessed depths.

### 3.4. Surface Contact Performance

**Figure 9:**
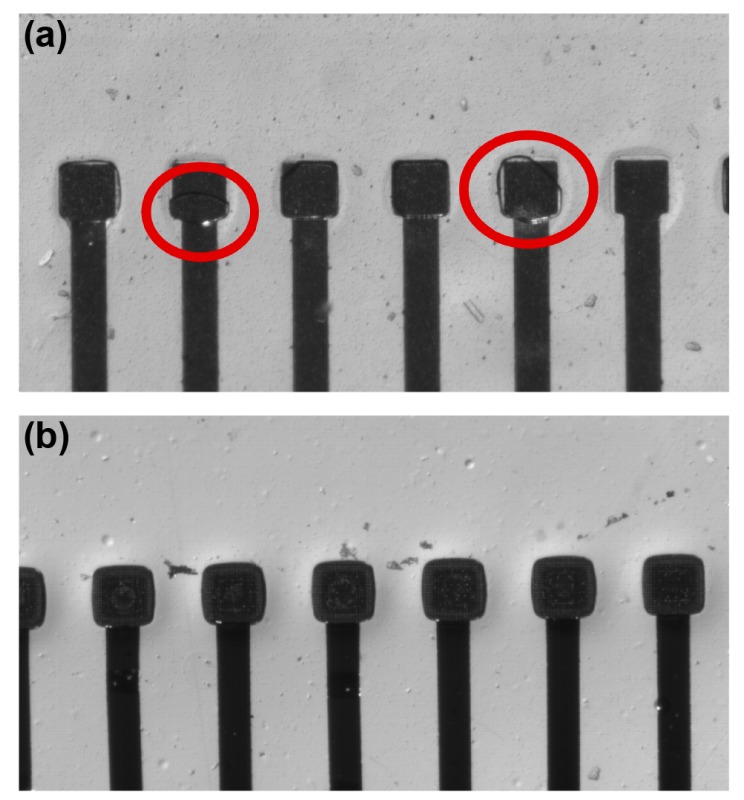
Optical microscope images for surface contact performance of plateau electrode. (**a**) An air bubble is trapped in the recessed electrode due to the hydrophobicity of the PDMS surface. (**b**) As there is no gap between the slide glass and electrodes, air bubbles are not seen on plateau electrodes.

We can observe the contact performance of the plateau electrode through the quality of interface between the electrode and a thin slide glass, as shown in Figure 9. An example of an air bubble trapped between the recessed electrode and a slide glass is shown in Figure 9b. Conventionally, the oxygen plasma treatment has been used to alleviate this trapped-air phenomenon. However, as previously described, surface modification via oxygen plasma is only a transient solution, and a new permanent scheme is necessary. There is no space to trap air in the case of the plateau electrode structure, as shown in Figure 9b. Thus, and it can not only be a permanent solution, but also improve the contact ability and signal recording performance. In addition, as the PDMS material exhibits high conformability and elasticity, so the performances of MEA can be further improved.

### 3.5. In Vivo Experiments

**Figure 10:**
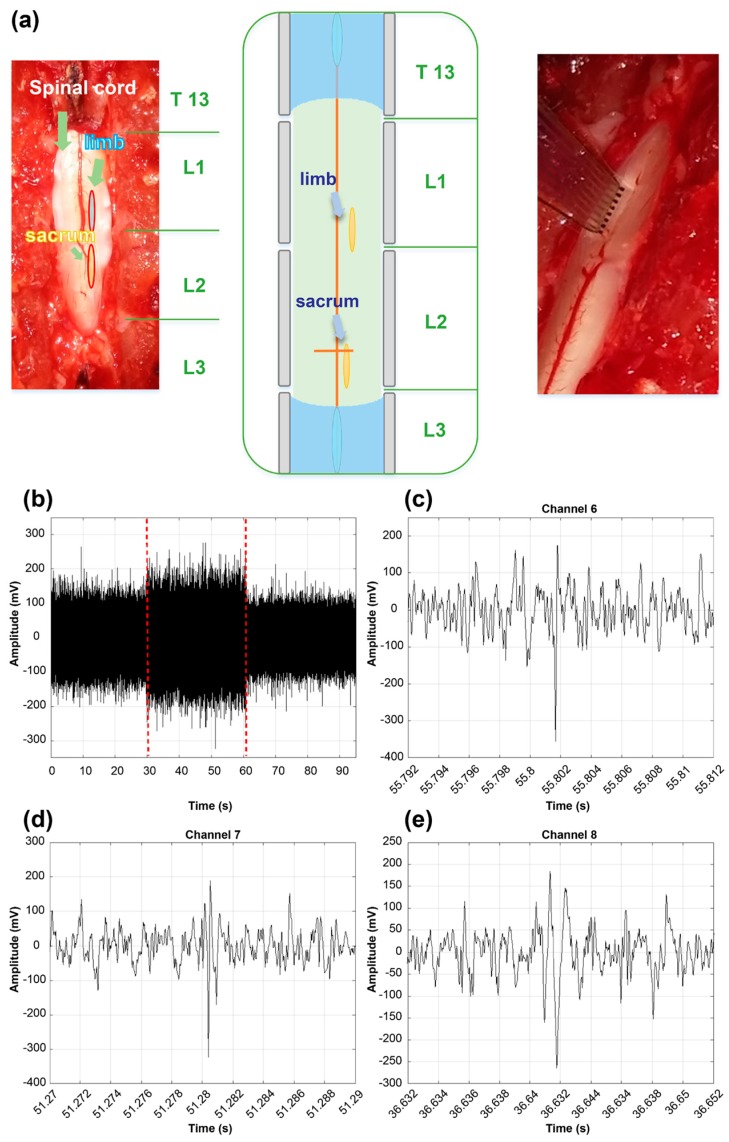
In vivo experiment. (**a**) In vivo placement of a PDMS-based MEA on a rat spinal cord. (**b**) Raw data from Channel 7. The period of the stimulus is indicated by the red dotted-lines. (**c**) An SSEPR from Channel 6. (**d**) An SSEPR from Channel 7. (**e**) An SSEPR from Channel 8.

The PDMS-based MEA was easily attached to the spinal cord, as shown in Figure 10a. Somatosensory evoked potentials (SSEPs) were observed as negative-first, biphasic responses after the stimulus on several channels in Figure 10c–e. Significant neural responses were recorded within the stimulus period. When using a recording electrode, the repopulation phenomenon is a very important factor in determining whether a biological signal can be recorded. In the spinal cord, which is a region without repopulation, we could not record a biological signal using recessed electrodes.

However, the plateau electrode could successfully record spinal cord signals. Figure 10a presents the recorded waveforms for 90 s from channel 7, and the red dotted line indicates the start and end of stimulus.

These experiments were conducted as proof-of-concept for the proposed fabrication. Future work can be to expand the fabrication method to various planar recording electrodes.

## 4. Conclusions

A PDMS-based plateau MEA has been successfully fabricated and its mechanical and electrical characteristics have been demonstrated. The PDMS material is flexible, chemically inert, and has a similar Young’s modulus as body tissues. It also satisfies properties that are necessary in the neural prosthesis field [18]. Moreover, the conformability and elasticity of PDMS are excellent compared to other polymers such as polyimide, parylene, and liquid crystal polymers (LCPs) [18,19]. Tests for cytotoxicity, extraction, and chronic toxicity have validated the biocompatibility of PDMS-based MEAs [11,16]. Recently, there is research to embed conductive materials such as Ag nanowires instead of depositing metal on PDMS, which have low surface energy and low bonding strength with other materials [33]. There is also research that utilizes the characteristics of PDMS with high elasticity, and use it as a touch sensor in a place with high movement [34]. A study has been done to fabricate transparent electrodes using the transparent characteristics of PDMS [35]. In this study we developed a method to fabricate electrode with surface-mounting structure by the molding technique. If we use this technique, a multilayer PDMS electrode can be developed, and the integration degree of the electrode can be increased. Therefore, we expect the PDMS-based plateau MEA to be an attractive alternative to conventional MEAs.

Two methods have been used widely for patterning metal films—wet-etching [12,17,20], and lift-off [14,18]. We have chosen the wet-etching method for its high resolution, stability, and yield of gold patterning. During a preliminary study of the lift-off method, we found that the photoresist film cracked during bake time due to a difference in the thermal expansion coefficients of PDMS and the photoresist. Photoresist cracking could be prevented by ensuring a slow temperature change of the sample, but in this case, a long period of time would be required to ensure that a reliable photoresist film is generated on the PDMS. To solve these problems, we performed photolithography on the sample after depositing metal on the PDMS layer. Thus, the metal deposited on the PDMS canceled out the thermal expansion of the PDMS and enabled patterning of the photoresist without cracks. While developing the photoresist, if the temperature of the developer is too low, the metal film could also become cracked when the photoresist cracks. Therefore, the developer (AZ MIF 300, AZ Electronic Material Corp., Branchburg, NJ, USA) should be used at approximately 30°. Furthermore, the adhesion between PDMS and metal was an important factor in achieving high resolution pattering. If the adhesion is low, the micro-pattern can be removed. By grafting HEMA onto PDMS, both the hydrophilicity retention time and adhesion force with the metal were improved.

The straight metal line on the PDMS substrate was easily damaged by tensile force, whereas a thin PDMS film can easily stretch. Serpentine-shaped metal patterns have been used to prevent metal line fractures caused by deformations and to provide stretchability upon tension or compression. Validation of the usage of serpentine metal lines was already performed successfully [32], and the results can be applied to the plateau electrode in order to solve the problem caused by tensile forces.

The impedance of the proposed plateau MEA was 150 kΩ at a frequency of 1 kHz. The proposed MEA was conformably placed over the spinal cord of a rat and neural activity was recorded. The conventional recessed MEAs could not record the neural signal due to the trapped air in the electrode. The proposed plateau electrode could record the neural signals reliably because the design prevents the air trapping phenomenon.

## Figures and Tables

**Table 1:** PDMS wet-etching rates using TBAF solution.

**NMP:TBAF (*v*/*v*)**	3:1 (25%)	5:1 (12%)	10:1 (9%)	15:1 (6%)
**Etching rate (μm/min)**	3.7	2.8	2	1.3

**Table 2:** Results of adhesion test with tapes 1 and 2.

Method	Time after treatment (h)	Adhesion test with tape 1	Adhesion test with tape 2	Contact angle (degrees)
No treatment	--	X	X	~105
Oxygen plasma	1	0%	0%	~10
	24	60%	80%	~92
HEMA	1	0%	0%	~12
	48	0%	0%	~20
	120	0%	0%	~35
